# Carbapenemase genes in clinical and environmental isolates of *Acinetobacter* spp. from Quito, Ecuador

**DOI:** 10.7717/peerj.17199

**Published:** 2024-04-25

**Authors:** Nicole Sotomayor, José Eduardo Villacis, Noela Burneo, Jorge Reyes, Sonia Zapata, Rosa de los Ángeles Bayas-Rea

**Affiliations:** 1 Escuela de Ciencias Biológicas, Pontificia Universidad Católica del Ecuador, Quito, Ecuador; 2 Centro de Referencia Nacional de Resistencia a los Antimicrobianos, Instituto Nacional de Investigación en Salud Pública-INSPI Dr. Leopoldo Izquieta Pérez, Quito, Ecuador; 3 Centro de Investigación para la Salud en América Latina (CISeAL), Pontificia Universidad Católica del Ecuador, Quito, Ecuador; 4 Facultad de Ciencias Químicas, Universidad Central del Ecuador, Quito, Ecuador; 5 Instituto de Microbiología, Colegio de Ciencias Biológicas y Ambientales, Universidad San Francisco de Quito, Quito, Ecuador

**Keywords:** Carbapenemases, Oxacilinases, Clinical isolates, River isolates, Quito, *Acinetobacter*, Ecuador, OXA-143, OXA-51, Carbapenem resistance

## Abstract

Carbapenem-resistant *Acinetobacter* spp. is associated with nosocomial infections in intensive care unit patients, resulting in high mortality. Although *Acinetobacter* spp. represent a serious public health problem worldwide, there are a few studies related to the presence of carbapenemases in health care facilities and other environmental settings in Ecuador. The main aim of this study was to characterize the carbapenem-resistant *Acinetobacter* spp. isolates obtained from four hospitals (52) and from five rivers (27) close to Quito. We used the disc diffusion and EDTA sinergy tests to determine the antimicrobial susceptibility and the production of metallo β-lactamases, respectively. We carried out a multiplex PCR of *gyrB* gene and the sequencing of partial *rpoB* gene to bacterial species identification. We performed molecular screening of nine carbapenem-resistant genes (*bla*_SPM_, *bla*_SIM_, *bla*_GIM_, *bla*_GES_, *bla*_OXA-23_, *bla*_OXA-24_, *bla*_OXA-51_, *bla*_OXA-58_, and *bla*_OXA-143_) by multiplex PCR, followed by identification using sequencing of *bla*_OXA_ genes. Our findings showed that carbapenem-resistant *A. baumannii* were the main species found in health care facilities and rivers. Most of the clinical isolates came from respiratory tract samples and harbored *bla*_OXA-23_, *bla*_OXA-366_, *bla*_OXA-72_, *bla*_OXA-65_, *bla*_OXA-70_, and *bla*_OXA-143-like_ genes. The river isolates harbored only the *bla*_OXA-51_ and probably *bla*_OXA-259_ genes. We concluded that the most predominant type of carbapenem genes among isolates were both *bla_OXA-23_* and *bla_OXA-65_* among *A. baumannii* clinical isolates.

## Introduction

*Acinetobacter* species are aerobic, Gram-negative pleomorphic, and non-lactose fermenting bacilli (Murray, Rosenthal & Pfaller, 2021) and they are ubiquitous in nature, including human skin (Almasaudi, 2016). In the past two decades, multidrug-resistant *Acinetobacter* spp. have become an important cause of health care-associated infections (HAIs), contributing to increase mortality rates in intensive care units (ICUs) (Castanheira, Mendes & Gales, 2023). Of the all *Acinetobacter* species, *A. baumannii* is predominant in clinical settings (Evans, Hamouda & Amyes, 2013; Silva et al., 2022) although non-*baumannii* species are also considered clinically important (Al Atrouni et al., 2016a). The World Health Organization (2017) elevated *A. baumannii* to the top of the list of critical pathogens. In Ecuador, *A*. *baumannii* was the main bacterium associated with HAIs in the ICU in 2010 and 2011 (Jiménez, 2013).

*Acinetobacter* species have high carbapenem resistance by the acquisition of genetic determinants of resistance through horizontal gene transfer (Castanheira, Mendes & Gales, 2023). The environment plays a key role in the spread of resistance to clinically relevant antibiotics (Singer et al., 2016); the spread of antibiotic resistance genes in river systems is a potential threat to public health (Wang et al., 2018). Environmental strains harbor antibiotic resistance mechanisms, serving as reservoirs for resistant elements and some of them could cause HAIs (Finley et al., 2013; Al Atrouni et al., 2016a). Horizontal gene transfer plays an important role in the transmission antibiotic resistance between non-pathogens and human pathogens (Wang et al., 2018). Worldwide, *A. baumannii* has increased the carbapenem resistance in around 90% in the last decade (Rosenthal et al., 2021). In Latin America, *Acinetobacter* spp. resistance to imipenem and meropenem varied from 8% to 89% (Pan American Health Organization, 2021). In 2010, Ecuador reported that 54% of *A. baumannii* strains were resistant to meropenem and 51% to imipenem (Labarca et al., 2016); however, the percentage of carbapenem resistant increased to 70% according to the Antimicrobial Resistance Surveillance Network of Ecuador (Satán et al., 2023).

The main mechanism of carbapenem resistance in *Acinetobacter* species is the production of serin-β-lactamases and metallo-β-lactamases enzymes (Castanheira, Mendes & Gales, 2023). In South America, the most common enzymes are oxacillinases (OXA) encoded by *bla*_OXA-23_, *bla*_OXA-72_, *bla*_OXA-51_, *bla*_OXA-58_, *bla*_OXA-235_, and *bla*_OXA-143_ genes (Escandón-Vargas et al., 2017; García-Betancur et al., 2021). There is scarce information about carbapenem-resistant strains from clinical and environmental settings in Ecuador. In the country, studies reported the presence of *bla*_NDM-1_, *bla*_OXA-23_, and *bla*_OXA-72_ genes in *A. baumannii* clinical isolates (Nuñez et al., 2016; Villacís et al., 2019). Thus, the main aim of this study was to determine the presence of carbapenem-resistant clinical and river *Acinetobacter* spp.

## Materials and Methods

### Clinical isolates

**Figure 1:**
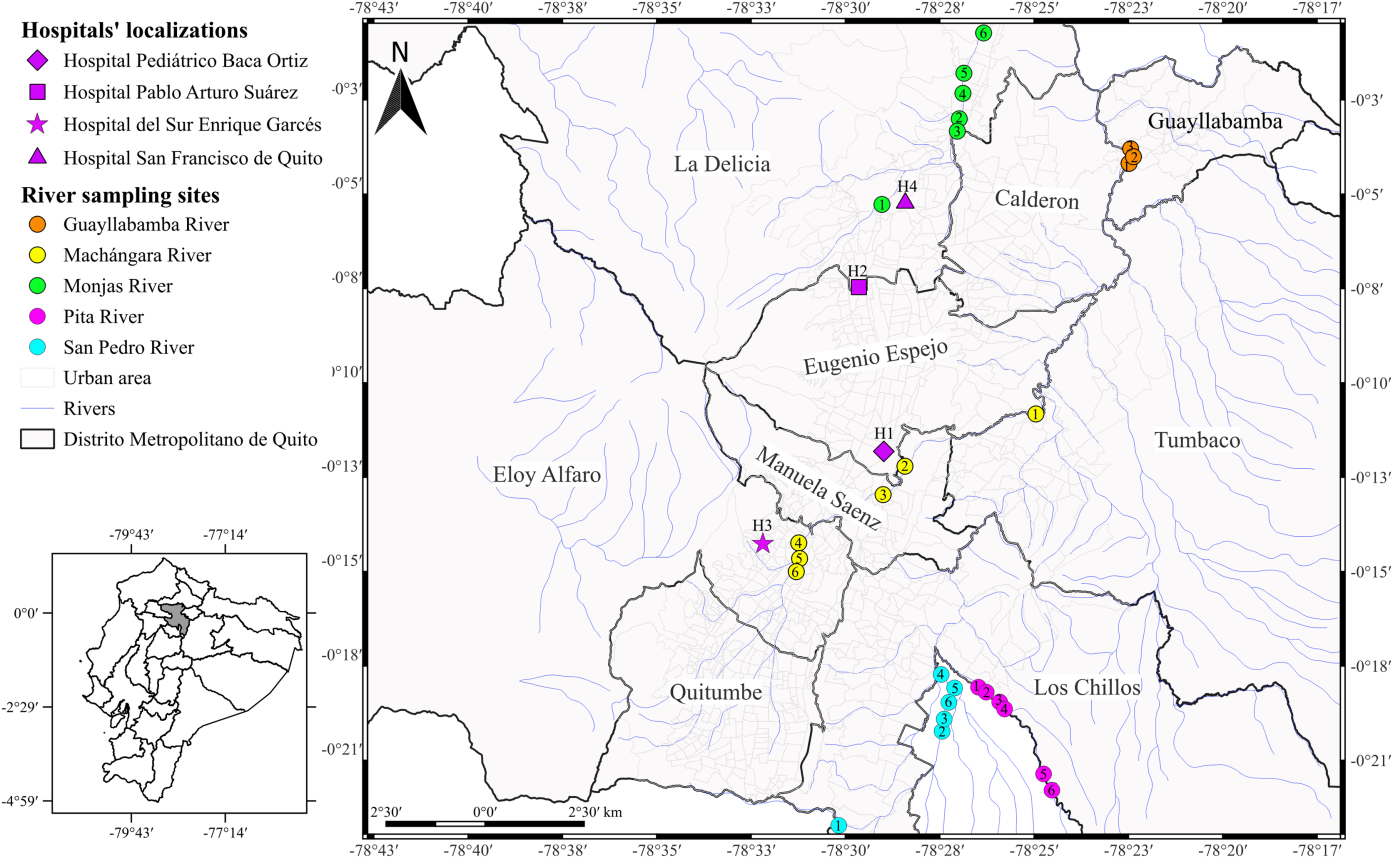
Map of the city of Quito showing sampling locations. *Acinetobacter* spp. clinical isolates recovered through the surveillance network from four hospitals in Quito. The hospitals’ localizations are represented by diamond (H1), square (H2), star (H3), and triangle (H4). The river sampling sites are represented by the circles, which indicates the GPS points where we collected the water sample. Rosa de los Ángeles Bayas-Rea made this map.

The National Reference Laboratory for Antimicrobial Resistance provided us a total of 52 *Acinetobacter* spp. clinical isolates recovered through the surveillance network from January to December 2015. As part of the standard procedure, the Reference Laboratory get back the *Acinetobacter* isolates from four different hospitals: Hospital Pediátrico Baca Ortiz (H1, *n* = 42), Hospital Pablo Arturo Suárez (H2, *n* = 5), Hospital del Sur Enrique Garcés (H3, *n* = 3), and Hospital San Francisco de Quito (H4, *n* = 2) in Quito (Fig. 1). The Reference Laboratory isolated *Acinetobacter* spp. from the following clinical samples: pleural fluid, cerebrospinal fluid, blood, urine, catheters, secretions, wounds, skin, abscesses, sputum, tracheal aspirates, and unidentified samples recovered from eleven units (ICU, general surgery, traumatology, burn unit, external consultation, neonatal unit, infectious diseases, pediatric unit, internal medicine, emergency, cardiology, and unidentified units). The data from each isolate are detailed in Table S1. The Reference Laboratory previously identified all isolates as *A. baumannii* based on VITEK 2 compact system® (Biomerieux, Marcy-I’Étoile, France).

We inoculated the frozen clinical isolates in sheep blood and MacConkey agars and incubated at 37 °C for 24 h. We used Gram staining, oxidase test, and catalase test to re-confirmed the identity of the isolates. We performed DNA extraction by boiling according to the protocol described by Cuaical et al. (2012); briefly, we boiled an overnight culture diluted in sterile water (500 μl) at 100 °C for 15 min, following centrifugation at 10,000 g for 2 min. We quantified the supernatant by spectrophotometry using the Nanodrop (Thermo Fisher Scientific, Waltham, MA, USA) and stored at −20 °C for subsequent amplification. We confirmed species identification by the amplification of partial RNA polymerase β (*rpoB*) gene and DNA gyrase subunit B (*gyrB*) gene according to La Scola et al. (2006) and Higgins et al. (2007, 2010a), respectively. The primers selected are detailed in Table 1.

**Table 1:** Primers used in this study for the identification of *Acinetobacter* spp. and detection of carbapenem-resistant genes.

Target gene	Sequence primer	Size (bp)	References
*gyrB*	Sp2F: GTTCTTGATCCGAAATTCTCG	490	Higgins et al. (2007)
	Sp4R: AACGGAGCTTGTCAGGGTTA		
	Sp4F: CACGCCGTAAGAGTGCATTA	294	
	Sp4R: AACGGAGCTTGTCAGGGTTA		
	Sp1F: GACAACAGTTATAAGGTTTCAGGTG	428	Higgins et al. (2010a)
	Sp1R: CCGCTATCTGTATCCGCAGTA		
	Sp3F: GATAACAGCTATAAAGTTTCAGGTGGT	194	
	Sp3R: CAAAAACGTACAGTTGTACCACTGC		
*rpoB*	Ac696F: TAYCGYAAAGAYTTGAAAGAAG	350	La Scola et al. (2006)
	Ac1093R: CMACACCYTTGTTMCCRTG		
	Ac1055F: GTGATAARATGGCBGGTCGT	450	
	Ac1598R: CGBGCRTGCATYTTGTCRT		
*bla*_SIM_	F: TACAAGGGATTCGGCATCG	570	Ellington et al. (2007)
	R: TAAGGCCTGTTCCCATGTG		
*bla*_GES_	F: ATGCGCTTCATTCACGCAC	863	Opazo et al. (2012)
	R: AACTCATCCTGAGCACGGAC		
*bla*_SPM_	F: AAAATCTGGGTACGCAAACG	271	Ellington et al. (2007)
	R: ACATTATCCGCTGGAACAGG		
*Bla*_GIM_	F: TCGACACACCTTGGTCTGAA	477	Ellington et al. (2007)
	R: AACTTCCAACTTTGCCATGC		
*bla*_OXA-23_	F: GATCGGATTGGAGAACCAGA	501	Woodford et al. (2006)
	R: ATTTCTGACCGCATTTCCAT		
*bla*_OXA-24_	F: GGTTAGTTGGCCCCCTTAAA	246	Woodford et al. (2006)
	R: AGTTGAGCGAAAAGGGGATT		
*bla*_OXA-51_	F: TAATGCTTTGATCGGCCTTG	353	Woodford et al. (2006)
	R: TGGATTGCACTTCATCTTGG		
*bla*_OXA-58_	F: AAGTATTGGGGCTTGTGCTG	599	Woodford et al. (2006)
	R: CCCCTCTGCGCTCTACATAC		
*bla*_OXA-143_	F: TTCTGTCAGTGCATGCTCATC	728	Opazo-Capurro (2014)
	R: CAGGCATTCCTTGCTTCATT		
*bla*_OXA-51_	F: CTTATAAGTCATATGAACATTAAAGC	975	Heritier, Poirel & Nordmann (2006)
	R: CTCTATAAAAAGGGATCCGGGCTA		
*bla*_OXA-24_	F: ATGAAAAAATTTATACTTCCTA TATTCAGC	825	Jeon et al. (2005)
	R: TTAAATGATTCCAAGATTTTCTAGC		
*bla*_OXA-23_	F: GATGTGTCATAGTATTCGTCG	1,058	Afzal-Shah, Woodford & Livermore (2001)
	R: TCACAACAACTAAAAGCACTG		

**Note:**

The primer name, the sequences of primer forward and reverse, the expected size, and reference where the sequences were obtained.

### River water collection and bacterial identification

We collected the water samples from five principal rivers: Monjas (MO, *n* = 6), Machángara (MA, *n* = 6), San Pedro (S, *n* = 6), Pita (P, *n* = 6), and Guayllabamba (G, *n* = 3) in March and April 2016 (Fig. 1 and Table S1). We chose those rivers for two reasons: (i) the four rivers are the main ones that run through Quito, all of them flow into the Guayllabamba River, and (ii) sewage is released into the city’s rivers without prior treatment. In addition, we chose the number of water collection points based on the accessibility and proximity to the urban center. We collected 100 ml of water samples at each river point and transported to the laboratory with ice packs according to Zhang et al. (2013) with few modifications. Subsequently, we centrifuged the water samples at 1,000 g for 10 min at room temperature, resuspended the pellets with 5 ml of fluid thioglycollate medium, and incubated at 37 °C overnight. We inoculated thirty microliters of cultured sample onto CHROMagar *Acinetobacter* plates with antimicrobial selective supplement (CR 202; CHROMagar Company, Paris, France) and incubated at 37 °C overnight. We selected all colonies with red pigmentation and identified through morphological and conventional biochemical tests used for the clinical isolates. We identified the positive potential isolates using VITEK-2 compact system® (Biomerieux, Marcy-I’Étoile, France). We confirmed the species identification by the amplification of DNA gyrase subunit B (*gyrB)* gene according to Higgins et al. (2007, 2010a).

### Antimicrobial susceptibility test

We use the disc diffusion method and the EDTA sinergy test to determinate the antimicrobial susceptibility and the production of metallo β-lactamases of clinical and river *Acinetobacter* spp. isolates (Clinical Laboratory Standards Institute, 2022). We spread a concentration corresponding to 0.5 of the McFarland scale of each isolate on Muller Hinton agar plates and incubated at 37 °C overnight. We included the following eleven antimicrobial susceptibility test discs (OXOID, Waltham, MA, USA): ampicillin/sulbactam (20 μg), ceftazidime (30 μg), ciprofloxacin (10 μg), imipenem (10 μg), meropenem (10 μg), gentamicin (10 μg), amikacin (30 μg), cefepime (30 μg), trimethoprim/sulfamethoxazole (25 μg), piperacillin/tazobactam (110 μg), and tobramycin (30 μg). We interpreted the resistance based on the susceptibility breakpoints of the Clinical Laboratory Standards Institute (2022). To assess the synergistic inhibition, we included an ethylenediaminetetraacetic (EDTA) disc (5 mg) with two carbapenem discs, imipenem (10 mg) and meropenem (10 mg), located at 2 cm from the center of the EDTA disc.

### Molecular identification of carbapenems-resistant genes

We performed two different multiplex PCRs to screen the presence of carbapenems-resistant genes including six genes encoding enzymes type serine β-lactamases (*bla*_GES_, *bla*_OXA-23_, *bla*_OXA-24_, *bla*_OXA-51_, *bla*_OXA-58_, and *bla*_OXA-143_) and three genes encoding metallo-β-lactamases (*bla*_SPM_, *bla*_SIM_, and *bla*_GIM_) using specific primers previously described (Ellington et al., 2007; Woodford et al., 2006) (Table 1). We achieved a PCR amplification in a final volume of 25 μl containing 1X of Master Mix Green GoTaq® (12.5 μl) (Promega, Madison, WI, USA), 10 μM of forward and reverse primers (0.5 μl), and 50 ng DNA (1 μl). To each PCR assay, we put positive, internal, and negative controls. Both multiplex PCR reaction conditions are detailed in Table 2. We identified all PCR products through electrophoresis in a 2% agarose gel stained with GelstarTM Nucleic Acid Gel Stain 10000 X staining (Lonza, Rockland, ME, USA) and visualized using Gel Doc XR system (Bio-Rad, Hercules, CA, USA).

**Table 2:** Single and multiplex PCR reaction conditions.

Carbapenemase class	Gene	Thermal cycling conditions					Reference
		Initial denaturation	35 cycles			Final extention	
			Denaturation	Annealing	Extention		
Multiplex PCR 1	*bla*_SPM_	94 °C for 5 min	94 °C for 30 s	52.8 °C for 40 s	72 °C for 50 s	72 °C for 10 min	Ellington et al. (2007)
	*bla*_GES_						
	*bla*_SIM_						
	*bla*_GIM_						
Multiplex PCR 2	*bla*_OXA-23_	94 °C for 5 min	94 °C for 30 s	53 °C for 30 s	72 °C for 30	72 °C for 10 min	Woodford et al. (2006)
	*bla*_OXA-24_						
	*bla*_OXA-51_						
	*bla*_OXA-58_						
	*bla*_OXA-143_						
Sequencing	*bla*_OXA-51_	94 °C for 2 min	94 °C for 1 min	52 °C for 30 s	72 °C for 1 min	72 °C for 10 min	Heritier, Poirel & Nordmann (2006)
	*bla*_OXA-23_	94 °C for 5 min	94 °C for 30 s	52 °C for 40 s	72 °C for 50 s	72 °C for 10 min	Afzal-Shah, Woodford & Livermore (2001)
	*bla*_OXA-24_	94 °C for 5 min	94 °C for 30 s	50 °C for 45 s	72 °C for 1 min	72 °C for 10 min	Jeon et al. (2005)
	*bla*_OXA-143_	94 °C for 5 min	94 °C for 30 s	53 °C for 30 s	72 °C for 30	72 °C for 10 min	Woodford et al. (2006)

**Note:**

The different thermal cycling conditions for various molecular analyses in this study.

We performed a conventional PCR to amplified the entire sequence of the following genes *bla*_OXA-23_, *bla*_OXA-24_, *bla*_OXA-51_, and *bla*_OXA-143_ genes using primers previously reported (Heritier, Poirel & Nordmann, 2006; Afzal-Shah, Woodford & Livermore, 2001; Jeon et al., 2005; Woodford et al., 2006) (Table 1), following the conditions detailed in Table 2. We cleaned the PCR products using exonuclease I and shrimp alkaline phosphatase (ExoSAP-IT®). We send both DNA strands of all positive *bla*_OXA_ genes for sequencing to Macrogen (Seoul, Korea). We aligned manually both forward and reverse sequences for editing and cleaning to obtain a single consensus sequence with the Clustal W program implemented in the MEGA 7 software (Kumar, Stecher & Tamura, 2016). We performed the identification of sequences obtained using a basic local alignment search tool (BLAST) (Altschul et al., 1997). We determined the number of sequences of *bla*_OXA-51_ and *bla*_OXA-23_ genes with DnaSP v.5.10 software (Rozas et al., 2010).

## Results

### Identification of *Acinetobacter* spp.

**Figure 2:**
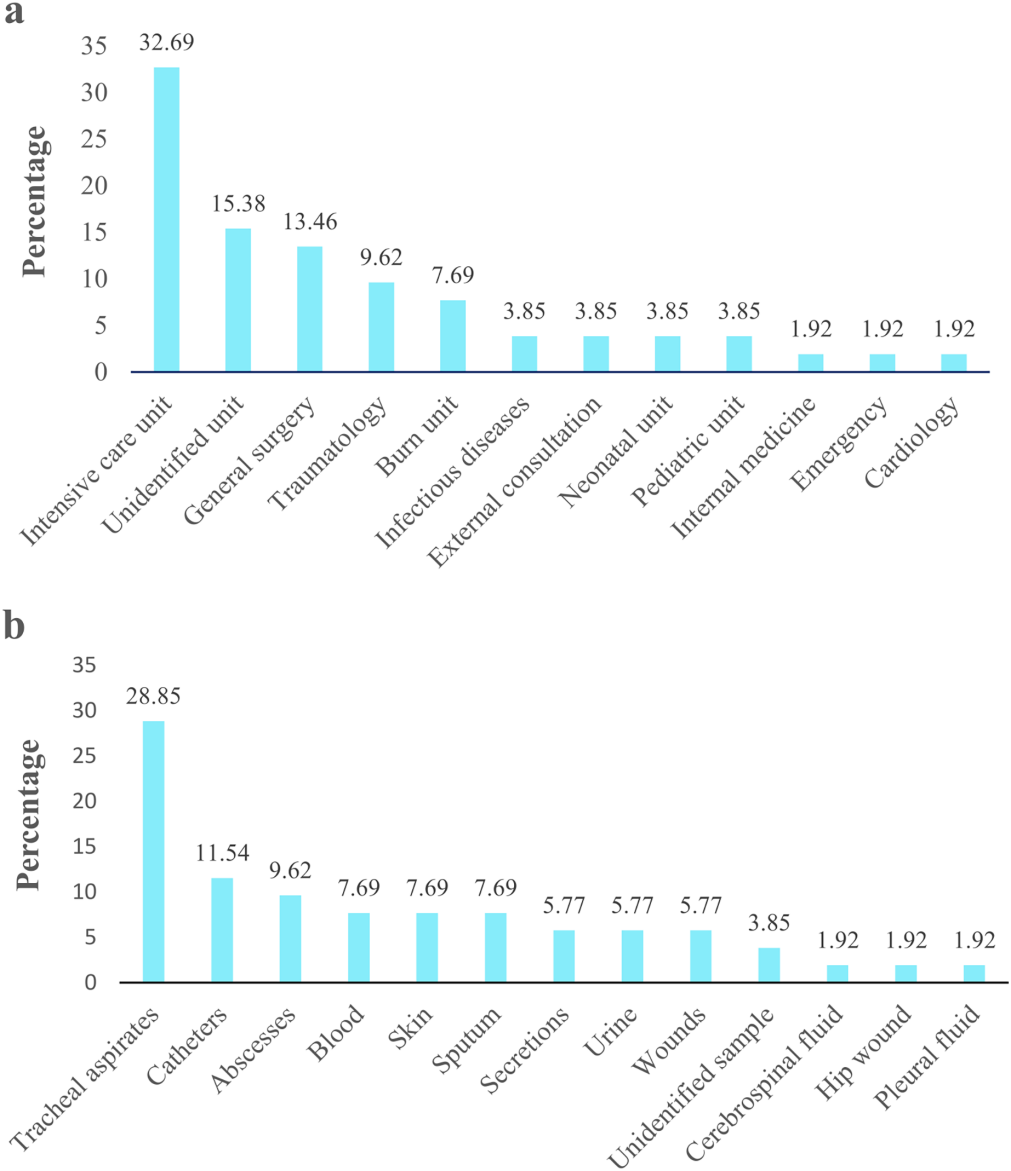
*Acinetobacter* spp. clinical isolates obtained from four hospitals in Quito. The percentage of *Acinetobacter* spp. clinical isolates. (A) Isolates obtained from different hospital service units. (B) Isolates recovered from clinical specimens.

*Acinetobacter* spp. clinical isolates originated mainly proceeded from pediatric population (77%; 40/52), followed by the adult population (19.2%; 10/52). Isolates derived principally from patients admitted in UCI 32.69% (17/52), followed by unidentified units 15.38% (8/52) and general surgery 13.46% (7/52) (Fig. 2A). According to the clinical specimens, the isolates came from tracheal aspirates 28.85% (15/52), followed by catheters 11.54% (6/52) and abscesses 9.62% (5/52) (Fig. 2B). The majority of clinical isolates were identified as *A. baumannii* (96.15% 50/52) and *A. pittii* (3.85%; 2/52) according *rpoB* gene. Only the identity of *A. pittii* (3.85%; 2/52) was confirmed with both markers, *rpoB* and *gyrB* genes. *A. baumannii* was found in all type of clinical specimen from all hospital units while *A. pittii* was found only in tracheal aspirates from UCI. The biochemical and molecular identification of each isolate is detailed in Tables S2 and S3. The nucleotide and amino sequences of partial *rpoB* gene are detailed in Informations S1 and S2. We submitted all *rpoB* sequences to GenBank with assigned accession numbers OP796738–OP796781.

A total of 27 water samples were collected from five rivers. Twenty-two samples presented suggestive *Acinetobacter* isolates, four samples (RMO3, RS5, RP4, and RG3) showed no growth, and one sample (RMO4) had isolates not related to *Acinetobacter* spp. We recovered twenty-six suggestive isolates from San Pedro (2), Machángara (4), Pita (1), and Monjas (3) rivers, of which only 38.5% (10/26) were identified as *Acinetobacter* spp. based on VITEK 2 compact® (Biomerieux, Marcy-I’Étoile, France) and as *A. baumannii* based on *gyrB* gene amplification. The remaining corresponded to other genera (*Pseudomonas aeruginosa*: 34.6%, *Stenotrophomonas maltophila*: 19.3%, *Burkkolderia cepacia*: 3.8%, and *Moraxella* group: 3.8%). The biochemical and molecular identification of each isolate is detailed in Tables S2 and S3.

### Phenotypic antimicrobial susceptibility

**Figure 3:**
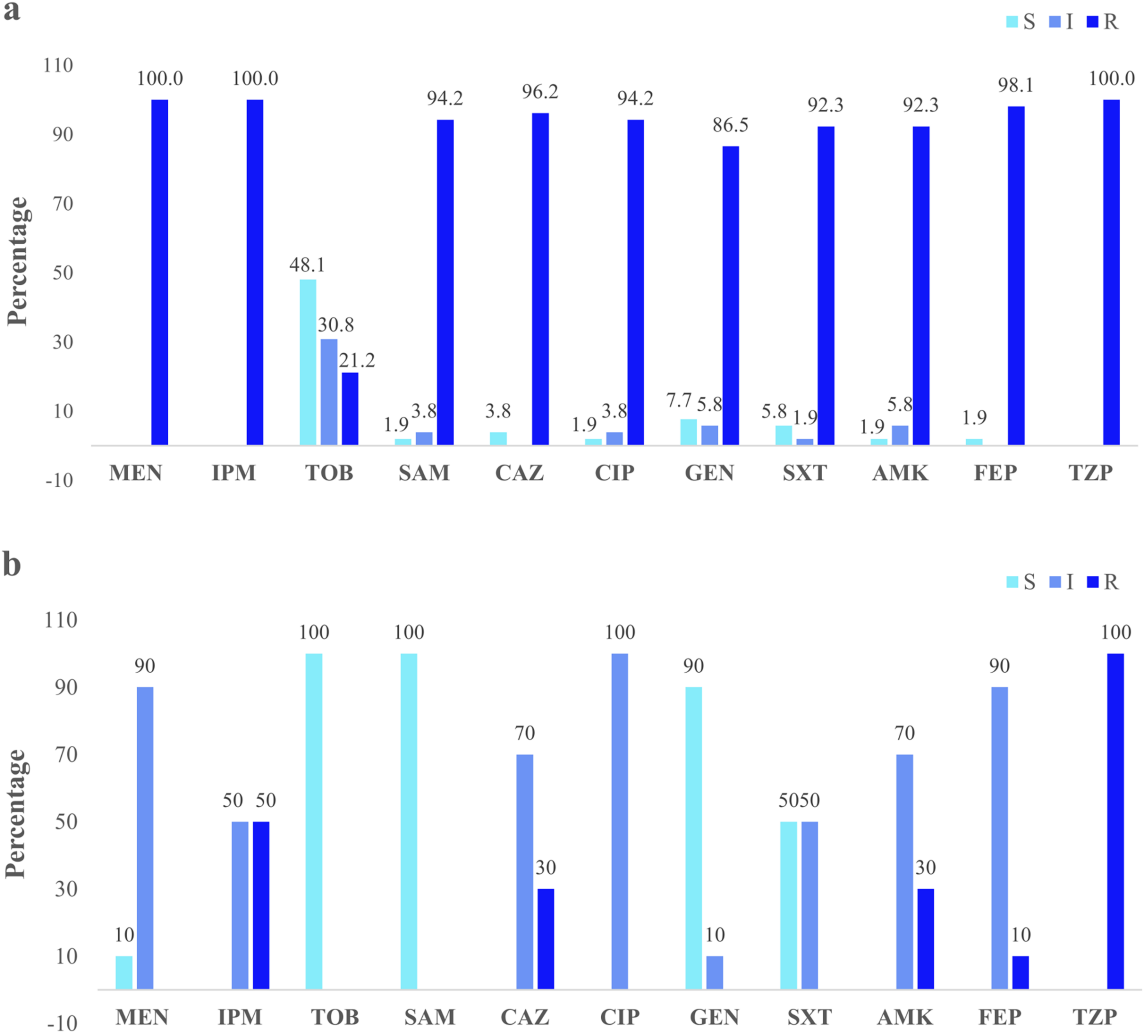
Clinical and environmental isolates susceptibility profile. The percentages of clinical isolates that were resistant, intermediate, or sensitive to each antibiotic. (A) Clinical isolates susceptibility profile, (B) river isolates susceptibility profile. Acronyms: CIP, ciprofloxacin; GEN, gentamycin; SXT, trimethoprim/sulfamethoxazole; AMK, amikacin; FEP, cefepime; MEN, meropenem; IMP, imipenem; TOB, tobramycin; SAM, ampicillin/sulbactam; CAZ, ceftazidime; TZP, piperacillin/tazobactam; R, resistant; I, intermediate; S, sensitive.

Clinical isolates presented the higher resistance (100%) to the three antibiotics: imipenem, meropenem, and piperacillin/tazobactam. Forty-eight percent were susceptible to tobramycin (Fig. 3A). The river isolates displayed an antimicrobial profile that varied between resistant and intermediate to eight antibiotics tested. Nevertheless, the main resistance was to piperacillin/tazobactam (100%). All of them were susceptible to tobramycin and ampicillin/sulbactam (100%) (Fig. 3B). According to the synergistic inhibition, both river and clinical isolates did not present metallo-β-lactamases enzymes. The antimicrobial susceptibility profile of the clinical and river isolates is detailed in Table S2.

### Molecular identification of carbapenem-resistance genes

According the screening of carbapenem-resistance genes, clinical isolates presented the following genes: *bla*_SIM_, *bla*_SPM_, *bla*_GES_, *bla*_OXA-23_, *bla*_OXA-24_, *bla*_OXA-51_, and *bla*_OXA-143_; whereas, seven river isolates showed *bla*_OXA-51_ gene (Table S3). The *bla*_SIM_ gene, co-harbored with the *bla*_OXA-72_ and *bla*_OXA-143_ genes, was the least frequently detected (1.9%; 1/52; 15-1175) in *A. pittii*. The *bla*_SPM_ gene, co-harbored with the *bla*_OXA-51_ and *bla*_OXA-23_ genes, was detected in 13.46% (7/52; 15-0122, 15-0591, 15-690, 15-780, 15-795, 15-853, 15-1,138) in *A. baumannii*. The *bla*_GES_ gene, co-harbored with the *bla*_OXA-51_, *bla*_OXA-23_, *bla*_OXA-72_, *bla*_OXA-143_, and *bla*_SIM_ genes was present in 11.53% (6/52) in *A. baumannii* (15-0669, 15-690, 15-853, 15-900) and *A. pittii* (15-691, 15-1,175). The *bla*_OXA-23_ gene, co-harbored with the *bla*_OXA-51_ gene, was found in 98.07% (51/52) present in *A. baumannii*. The *bla*_OXA-24_ gene, co-harbored with both *bla*_OXA-23_ and *bla*_OXA-51_ genes, was present in 9.6% (5/52) in *A. baumannii* (15-0659, 15-0985, 15-1064, and 15-1367) and in *A. pittii* (15-0691). The *bla*_OXA-143_ gene was the least frequently detected (1.9%; 1/52; 15-1,175); this gene was co-harbored with the *bla*_OXA-24_ gene in *A. pittii*.

### Carbapenem-resistance genes sequences analysis

We reported 47 *bla*_OXA-51_ sequences: 42 complete sequences of 813 bp and five partial sequences of 340 bp from *A. baumannii* clinical isolates. Of *bla*_OXA-51_ sequences, we identified two genes encoding OXA-65 (*n* = 44), OXA-70 (*n* = 2) and one unidentified (MF594746). Forty complete sequences (MF594725–MF594728, MF594730–MF594737, MF594735, MF594739, MF594741–MF594745, OP554146–OP554168) and four partial sequences (MF594729, MF594734, MF594736, and MF594737) shared 100% of nucleotide identity with *bla*_OXA-65_ of *A. baumannii* (GenBank accession number CP033869.1); whereas two entire sequences (MF594740 and MF594747) shared 100% nucleotide identity with *bla*_OXA-70_ of *A. baumannii* (GenBank accession number NG_049811.1).

We found 48 *bla*_OXA-23_ sequences: 42 complete sequences of 820 bp and six partial sequences of 471 bp from *A. baumannii* clinical isolates. Of *bla*_OXA-23_ sequences, we identified two genes encoding OXA-23 (*n* = 38) and OXA-366 (*n* = 10). Thirty-two entire sequences (MF594756–MF594759, MF594762–MF594767, MF594769, MF594772–MF594777, OP554169, OP554170, OP554172–OP554178, OP554180–OP554182, OP554185–OP554187, OP554189, OP554191–OP554193) and six partial sequences (MF594755, MF594760, MF594761, MF594768, MF594770, and MF594771) shared 100% of nucleotide identity with *bla*_OXA-23_ of *A. baumannii* (GenBank accession number CP110468.1). Ten complete sequences (MF594757, MF59474–MF59476, OP554171, OP554179, OP554183, OP554184, OP554188, and OP554190) shared 100% nucleotide identity with *bla*_OXA-366_ of *A. baumannii* (GenBank accession number NG_049659.1).

We reported five *bla*_OXA-24/40_ entire sequences of 817 bp (MF594778, MF594779, MF594781, MF594782, and MF594783) and one partial sequence of 204 bp (MF594780). Of *bla*_OXA-24/40_ sequences, we identified one gene encoding OXA-72 present in three *A. baumannii* and two *A. pitti* clinical isolates that shared 100% of nucleotide identity with *bla*_OXA-72_ of *A. baumannii* (GenBank accession number MN495626) and also *A. pittii* (GenBank accession number MN481287.1).

**Figure 4:**
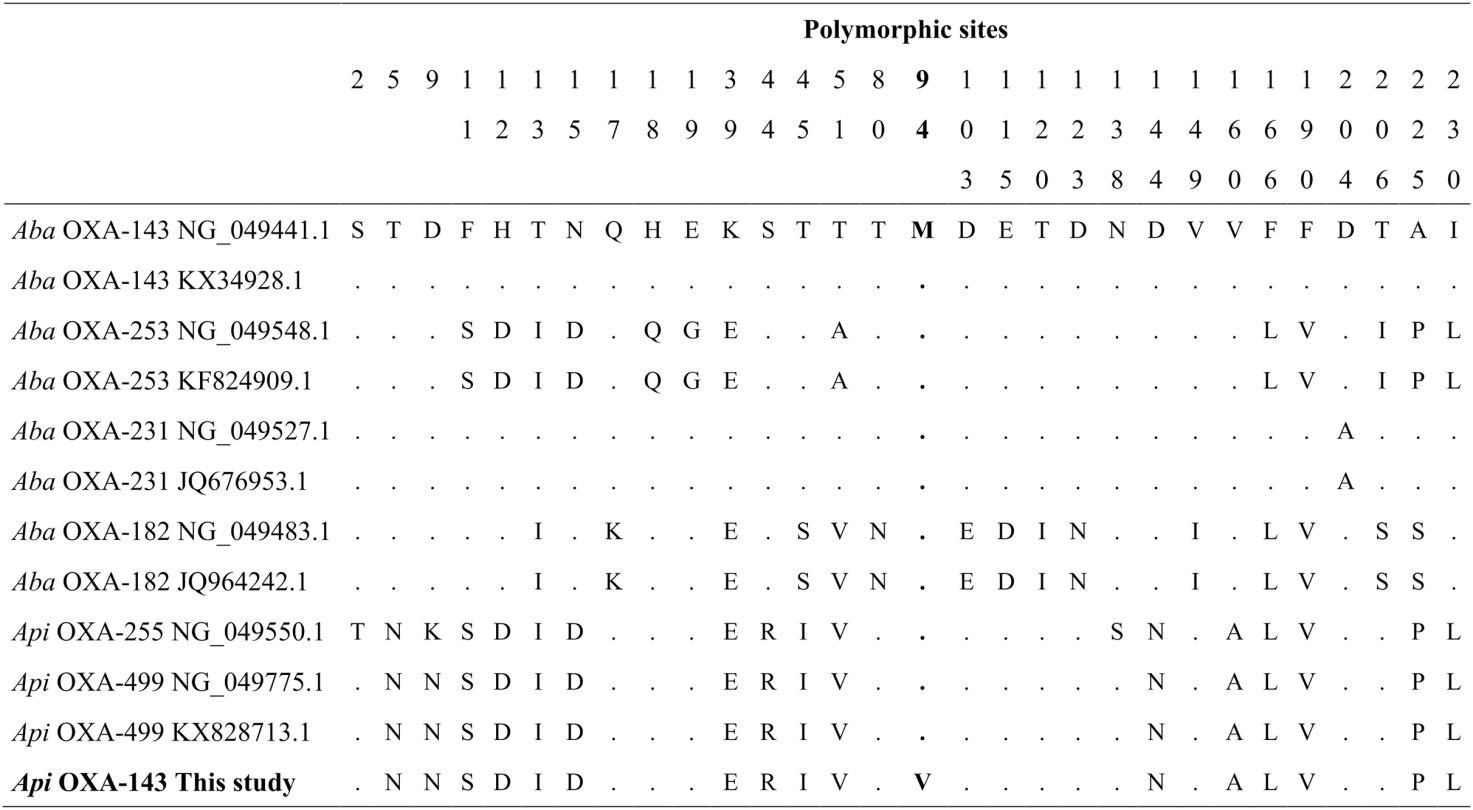
Alignment of OXA-143 amino acid sequences. The polymorphic sites at the amino acid sequence level. The identity of the sequences was compared to the OXA-143 variant (GenBank protein number access WP_063861042.1 or its nucleic number access NG_049441.1). Numbers in the heading row indicate the base pair position of the polymorphic sites. Dots (.) show the identity with the Aba OXA-143. Acronyms: Aba, *Acinetobacter baumannii*; Api, *A. pittii*.

Sequencing of the *bla*_OXA-143_ PCR product obtained from *A. pitti* clinical isolates identified gene encoding OXA-499. Our *bla*_OXA-143_ sequence (MF594724) shared 99% nucleotide and amino acid identity with *bla*_OXA-499_ of *A. pittii* (GenBank accession numbers NG_049775.1 and ALM96709.1). The nucleotide sequence presented one specific change in nucleotide 280, first position of the codon (GTG) (ATG), resulting in a valine (V) to methionine (M) change in position 94 (Fig. 4).

Sequencing of the *bla*_OXA-51_ PCR products obtained from *A. baumannii* river isolates identified two genes encoding OXA-65 and OXA-259. Four partial sequences (MF594748–MF594751) from three rivers (Pita, Machángara, and San Pedro) shared 100% of nucleotide identity with *bla*_OXA-65_ of *A. baumannii* (GenBank accession number OL961415.1) and three partial sequences (MF594752–MF594754) from Machángara River shared 100% of nucleotide identity with *bla*_OXA-259_ of *A. baumannii* (GenBank accession number CP053098.1).

The nucleotide and amino sequences of *bla*_OXA_ genes are detailed in the Informations S3 to S10. We submitted all sequences to GenBank with assigned accession numbers MF594724–MF594783, OP554146–OP554193.

## Discussion

### *Acinetobacter* spp. in clinical and river isolates

Our findings indicated that *Acinetobacter baumannii* is the most common species found in clinical isolates from tracheal aspirates from ICU. Similarly, studies reported high rates of *baumannii* species in ICU samples elsewhere (Qin et al., 2021; Bakhshi et al., 2022). Previous Ecuadorian studies reported high percentages of *A. baumannii* from respiratory tract samples, bronchial secretions, or tracheal aspirates (Jiménez, 2013; Nuñez et al., 2016; Villacís et al., 2019). On the other hand, we found *A. baumannii* in the most polluted river environments, Machángara and Monjas rivers (Yungán, 2010; Roldós, 2015). Similar, some studies found these bacteria in river water as well as sewage water (Hrenovic et al., 2016; Kisková et al., 2023). Pathogens of clinical origin as *A. baumannii* can reach aquatic environments as a result of different human activities, such as sewage water discharge directly into the river without pretreatment (Silva et al., 2016). *Acinetobacter* spp. survive in wastewater contributing to the spread clinical antibiotic resistance genes in aquatic systems (Kisková et al., 2023; Tobin et al., 2024). Particularly, hospital sewage water is considered a major source of multidrug-resistant pathogenic bacteria and antibiotic residues (Zhang et al., 2013). Contrary, we did not isolate any *Acinetobacter* spp. from the Guayllabamba River probably due to the distance (2 km), the collection points were far away from the wastewater reservoirs of urban areas and the bacterial concentrations decreases and distribution changes (Zhang et al., 2009).

### Carbapenem-resistant *Acinetobacter* spp.

In our study, the clinical and environmental *Acinetobacter* isolates showed different susceptibility profiles. Clinical isolates exhibited a high rate of resistance to imipenem and meropenem; while, river isolates displayed a low resistance only to imipenem. In general, clinical isolates have high resistance to both antibiotics (Cortivo et al., 2015). The percentage of resistance found in this study was higher compared with an Ecuadorian study (Jiménez, 2013). River isolates are usually more sensitive compared to clinical isolates. The difference in the patterns of resistance found can be due to various factors such as, biological conditions, virulence factors, epidemiology, or place of origin (Karmostaj, Najar & Salmanian, 2013). In general, it is expected that the antibiotic concentration is lower in aquatic environments than in clinical environments (Zhang et al., 2009; Zhang et al., 2013). In clinical environments, antibiotics (in particular broad-spectrum antibiotics) are supplied continuously, promoting selective pressure in clinical isolates. Following this, the transmission of genetic determinants among clinical isolates could take place through horizontal gene transfer (Zhang et al., 2009; Castanheira, Mendes & Gales, 2023). Nonetheless, the possibility of clonal dissemination as a mean of increasing antibiotic resistance, should not be discarded. This situation generates a faster antibiotic resistance evolution in clinical isolates than in environmental isolates (Zhang et al., 2013). However, the susceptibility results of environmental samples should be treated with caution, as these profiles were based on clinical standards (CLSI) and a small sample size (*n* = 10).

### Carbapenemases genes in *Acinetobacter* spp.

The high percentage of carbapenem-resistance found in clinical isolates may be increased due to the presence of *bla*_OXA-51_, *bla*_OXA-23_, *bla*_OXA-72_, and *bla*_OXA-143_ genes. According to some studies, the association of *bla*_OXA-23_ and *bla*_OXA-51_ genes as the main cause for carbapenem resistance (*i.e*., Martínez & Mattar, 2012; Teixeira et al., 2013; Cortivo et al., 2015). The *bla*_OXA-72_ gene increases its resistance activity in the presence of imipenem and ampicillin/sulbactam (Kuo et al., 2013). The *bla*_OXA-143_ and *bla*_OXA-72_ genes in association with intrinsic mechanisms as the alteration of the of the outer membrane proteins promotes a high resistance to meropenem (Mostachio et al., 2012; Sen & Joshi, 2015). The low percentage of carbapenem-resistance found in environmental isolates may be explained by the absence of *bla*_OXA_ genes. It is necessary the presence of insertion elements to promote overexpression of the *bla*_OXA-51_ gene since this gene it generates a weak resistance (Higgins, Zander & Seifert, 2013; Teixeira et al., 2013; Sen & Joshi, 2015). The *bla*_OXA-51_ overexpression would be linked to the resistance to imipenem in *A. baumannii* river isolates (Martínez & Mattar, 2012; Evans, Hamouda & Amyes, 2013). It would be important to know about possible IS*Aba1* insertions upstream of the gene or alternative genes conferring resistance in the five isolates with resistance phenotype.

We found a high percentage of *bla*_OXA-23_ and *bla*_OXA-51_ genes in *A. baumannii* and *A. pittii* clinical isolates. The high percentage of *bla*_OXA-23_ gene found in *A. baumannii*, is related to its distribution worldwide since a single sequence was found in the study. The *bla*_OXA-23_ gene distribution has been associated with a clonal spread (Higgins et al., 2010b; Labarca et al., 2016). In the other hand, *bla*_OXA-51_ gene has been reported principally in *baumannii* species (Lee et al., 2012; Teixeira et al., 2013; Périchon et al., 2014). Since *bla*_OXA-51_ gene can be found in the chromosome (Poirel, Naas & Nordmann, 2010), some authors consider it as an intrinsic marker for *A. baumannii* identification (Higgins et al., 2010b; Périchon et al., 2014). However, the gene may be in transposons and plasmids (Liu et al., 2015; Wang et al., 2015; Sennati et al., 2016). Its dissemination among other *Acinetobacter* species from different environments is possible, suggesting that the dissemination may not be by clonal strains (See Labarca et al., 2016).

Our findings detected two different *bla*_OXA-51_ variants OXA-65 and OXA-70 were circulating in the four hospitals; while, in rivers probably are OXA-65 and OXA-259, suggesting that the genes were under selective pressure. Similarly, previous studies OXA-65 was reported in *A. baumannii* clone ST79 from Guayaquil (Rodríguez et al., 2016). In Latin America, is one of the variants found in *A. baumannii* in Brazil, Argentina, Paraguay, Mexico, and Honduras (Rodríguez et al., 2016; Nodari et al., 2020; Graña-Miraglia et al., 2020). There is not report about the presence of OXA-70 neither OXA-259 variant in South America. The information about OXA-70 variant is scare worldwide. OXA-70 has been reported in Hong Kong, Malaysia, Ghana, and Honduras (Alattraqchi et al., 2020; Ayibieke et al., 2020; Brown & Amyes, 2005; Galac et al., 2020). OXA-70 belong to the most diverse collection of class D carbapenemases, subgroup 3 that is the major mechanism of carbapenem resistance in *A. baumannii* (Brown & Amyes, 2005). OXA-259 variant has been reported in *A. baumannii* clinical isolates from China, Tanzania, and Thailand (Jia et al., 2019; Moyo et al., 2021; Chukamnerd et al., 2022) as well in bulk tank milk in Germani (Sykes et al., 2023). The variant OXA-259 is located on the chromosome of *A. baumannii* and constitute an intrinsic oxacillinase genes with low-level carbapenemase activity that naturally occur (Chukamnerd et al., 2022), due there is a not ISA_balike_ elements (Fedrigo et al., 2022). To the best of our knowledge, this is the first report of the OXA-70 and OXA-259 in *A. baumannii* in Ecuador.

Our findings showed the presence of *bla*_OXA-366_ gene in both species *A. baumannii* and *A. pittii* clinical isolates from Ecuador. The information about this variant is scare. GenBank previously reported a sequence of *bla*_OXA-366_ in *A. baumannii* from USA. Whereas the OXA-366 variant has been reported also in *A. baumannii* clinical isolates in Iran (Bahador et al., 2015). To the best of our knowledge, this is the first report of the OXA-366 in *A. baumannii* and *A. pittii* in South America.

The *bla*_OXA-72_ gene co-harbored with *bla*_OXA-23_ and *bla*_OXA-51_ genes was identified in low levels in *A. baumannii* and *A. pittii*. This variant was documented previously in *A. baumannii* clinical isolates from Guayaquil (Nuñez et al., 2016; Rodríguez et al., 2016) and from neighboring countries Colombia, Peru, and Brazil (Saavedra et al., 2014; Saavedra et al., 2017; Werneck et al., 2011; de Sá Cavalcanti et al., 2013; Levy-Blitchtein et al., 2018; Nodari et al., 2020) and in *A. pittii* from Colombia (Montealegre et al., 2012), Brazil (Brasiliense et al., 2019), European and Asian countries (Al Atrouni et al., 2016b; Bonnin et al., 2014). The presence of this gene in two species may be due to the *bla*_OXA-72_ gene spreads through horizontal gene transfer mediated by plasmids (Montealegre et al., 2012; Kuo et al., 2013; Nuñez et al., 2016; Rodríguez et al., 2016). To the best of our knowledge, this is the first report of the *bla*_OXA-72_ gene in *A. pittii* in Ecuador.

This is also the first report of the *bla*_OXA-143_ gene in *A. pittii* clinical isolates from Ecuador. In South America, this gene and its variants (OXA-231, OXA-253, and OXA-255) have been reported in *A. baumannii* clinical isolates in Brazil (Higgins et al., 2009; Gionco et al., 2012; Girlich et al., 2014), Perú (Levy-Blitchtein et al., 2018), and Colombia (Saavedra et al., 2017). The variant found in this study is very different from OXA-143 variants reported in *A. baumannii* from Brazil, Honduras, and Korea (Higgins et al., 2009; Zander et al., 2014; Kim et al., 2010). Interestingly, the sequence alignment showed a high similarity between our variant with two OXA-143 variants (OXA-499 and OXA-255) described to *A. pittii* in Korea (D’Souza et al., 2017) and United States (Zander et al., 2014). In particular, the difference between the variant reported in this study differs by a change in amino acid sequence with OXA-499 reported by D’Souza et al. (2017). To the best of our knowledge, this is the first report of a variant similar to OXA-499 in *A. pittii* in South America.

## Conclusions

Overall, our results showed that the most of the *Acinetobacter* spp. clinical isolates came from tracheal aspirates from ICU. Our findings showed that carbapenem-resistant *A. baumannii* were the main species found in the study. We reported the presence of *bla*_OXA-65_, *bla*_OXA-70_, *bla*_OXA-72_, *bla*_OXA-23_, *bla*_OXA-366_, and *bla*_OXA143_-carrying multidrug-resistant genes in *baumannii* and no *baumannii* species. We report a new possible variant of OXA-143 circling in different hospitals in Quito and the possible presence of OXA-259 in aquatic environment. The presence of *A. baumannii* in the rivers increases the concern, since the resistance can be disseminated, polluting water sources, and promoting community acquired outbreaks.

To our knowledge, this is the first study that has characterized the carbapenemases genes in *Acinetobacter* spp. carbapenem-resistant isolates from four hospitals and five rivers in Quito. No *bla*_OXA-65_, *bla*_OXA-70_, *bla*_OXA-72_, *bla*_OXA-23_, *bla*_OXA-366_, or *bla*_OXA143_-carrying multidrug-resistant *baumanii* and no *baumannii* species have been described in Quito so far.

## Supplemental Information

10.7717/peerj.17199/supp-1 Supplemental Information 1 Clinical and River isolates data. All samples correspond to Zone 9, which represents the code for Quito. The table details the hospital code, INSPI isolate code, geographical coordinates age, sex, sample type, and hospital unit of the patients to which each sample corresponds. In addition, the table indicated the river data, id code, geographical coordinates, and river name. Acronyms: NA: not available.

10.7717/peerj.17199/supp-2 Supplemental Information 2 Biochemical results and antibiotic susceptibility profile of *Acinetobacter* spp. NA: Not applied, Aba: *A. baumannii*, Aha: *A. haemolyticus*, ABC: *A. baumannii/calcoaceticus* complex, ng/µl: nanograme/microlitre, Y: yes, N: not. Biochemical results are expressed as positive (+) and negative (−). The antibiotic susceptibility profile are expresed as Intermediate (I), Sensistive (S), and Resistance (R).

10.7717/peerj.17199/supp-3 Supplemental Information 3 Molecular results of *Acinetobacter* spp. obtained by amplification and sequencing of different genes. *A. baumannii* was identified by the amplification of both products, 490 bp and 294 bp, and *A. pittii* with a product of 194 bp of *gyrB* gene. The species identification was corroborate by sequencing on partial *rpo* gene. PCR results are expressed for carbapenen resistance genes as positive (+) and negative (−). The table details the GenBank number acccess of sequences obtained in this study.

10.7717/peerj.17199/supp-4 Supplemental Information 4 Partial rpoB gene nucleotide sequence matrix.

10.7717/peerj.17199/supp-5 Supplemental Information 5 Partial rpoB gene protein sequence matrix.

10.7717/peerj.17199/supp-6 Supplemental Information 6 *bla_OXA-23_* nucleotide sequences matrix.

10.7717/peerj.17199/supp-7 Supplemental Information 7 *bla_OXA-24_* nucleotide sequences matrix.

10.7717/peerj.17199/supp-8 Supplemental Information 8 *bla_OXA-51_* nucleotide sequences matrix.

10.7717/peerj.17199/supp-9 Supplemental Information 9 *bla_OXA-143_* nucleotide sequence.

10.7717/peerj.17199/supp-10 Supplemental Information 10 OXA-23 amino acid sequences matrix.

10.7717/peerj.17199/supp-11 Supplemental Information 11 OXA-24 amino acid sequences matrix.

10.7717/peerj.17199/supp-12 Supplemental Information 12 OXA-51 amino acid sequences matrix.

10.7717/peerj.17199/supp-13 Supplemental Information 13 OXA-143 amino acid sequence.

## Additional Information and Declarations

### Data Availability

The following information was supplied regarding data availability: The sequences are available at GenBank: MF594724–MF594783, OP796381.1–OP796781.1, OP554146.1–OP554193.1. The raw measurements are available in the Supplemental Files.
