# Supplementary material for: Carbapenemase genes in clinical and environmental isolates of Acinetobacter spp. from Quito, Ecuador
Source: PeerJ. 2024 Apr 25;12:e17199. doi: 10.7717/peerj.17199 (PMC11056107; doi:10.7717/peerj.17199)
Supplement: Supplemental Information 1 — All samples correspond to Zone 9, which represents the code for Quito. The table details the hospital code, INSPI isolate code, geographical coordinates age, sex, sample type, and hospital unit of the patients to which each sample corresponds. In addition, the table indicated the river data, id code, geographical coordinates, and river name. Acronyms: NA: not available. [file peerj-12-17199-s001.docx]

| **Hospital/River code** | **Isolated code** | **Geographical Coordinates** | **Age** | **Sex** | **Sample type** | **Hospital units/River name** |
| --- | --- | --- | --- | --- | --- | --- |
| H1 | 15-0014 | -0.202333 -78.486194 | 2 years | Female | Urine | Emergency |
| H3 | 15-0107 | -0.24425 -78.541083 | 60 years | Male | Unidentified | Unidentified |
| H1 | 15-0115 | -0.202333 -78.486194 | 5 months | Male | Tracheal aspirates | Intensive care unit |
| H1 | 15-0117 | -0.202333 -78.486194 | 2 years | Female | Secretions | Unidentified unit |
| H2 | 15-0122 | -0.127778 -78.49750 | 39 years | Female | Abscesses | General surgery |
| H2 | 15-0176 | -0.127778 -78.49750 | 45 years | Male | Secretions | Internal medicine |
| H1 | 15-0181 | -0.202333 -78.486194 | 12 years | Male | Wounds | Infectious diseases |
| H1 | 15-0184 | -0.202333 -78.486194 | 15 years | Female | Tracheal aspirates | Intensive care unit |
| H1 | 15-0252 | -0.202333 -78.486194 | 15 years | Female | Tracheal aspirates | Intensive care unit |
| H1 | 15-0255 | -0.202333 -78.486194 | 7 years | Female | Catheters | Pediatric unit |
| H1 | 15-0259 | -0.202333 -78.486194 | 1 year | Male | Urine | Unidentified unit |
| H1 | 15-0499 | -0.202333 -78.486194 | 52 days | Male | Catheters | Intensive care unit |
| H1 | 15-0500 | -0.202333 -78.486194 | 1 year | Female | Skin | Burn unit |
| H1 | 15-0502 | -0.202333 -78.486194 | 2 years | Female | Tracheal aspirates | Neonatal unit |
| H1 | 15-0540 | -0.202333 -78.486194 | 14 years | Male | Tracheal aspirates | Intensive care unit |
| H1 | 15-0577 | -0.202333 -78.486194 | 6 years | Male | Tracheal aspirates | General surgery |
| H1 | 15-0578 | -0.202333 -78.486194 | 13 years | Male | Blood | General surgery |
| H1 | 15-0579 | -0.202333 -78.486194 | 13 years | Male | Catheters | General surgery |
| H1 | 15-0580 | -0.202333 -78.486194 | 6 years | Female | Urine | Intensive care unit |
| H1 | 15-0581 | -0.202333 -78.486194 | 8 years | Male | Blood | Intensive care unit |
| H1 | 15-0582 | -0.202333 -78.486194 | 1 year | Male | Tracheal aspirates | Intensive care unit |
| H1 | 15-0589 | -0.202333 -78.486194 | 7 years | Female | Skin | Burn unit |
| H1 | 15-0590 | -0.202333 -78.486194 | 1 year | Male | Central catheters | General surgery |
| H2 | 15-0591 | -0.127778 -78.49750 | 82 years | Male | Sputum | Traumatology |
| H1 | 15-0608 | -0.202333 -78.486194 | 14 years | Male | Catheters | Intensive care unit |
| H1 | 15-0610 | -0.202333 -78.486194 | 2 years | Male | Abscesses | Burn unit |
| H1 | 15-0659 | -0.202333 -78.486194 | 4 years | Female | Secretions | Traumatology |

**Continue…**

| **Hospital/River code** | **Isolated code** | **Geographical Coordinates** | **Age** | **Sex** | **Sample type** | **Hospital units/River name** |
| --- | --- | --- | --- | --- | --- | --- |
| H1 | 15-0669 | -0.202333 -78.486194 | 1 year | Male | Blood | Unidentified unit |
| H1 | 15-0687 | -0.202333 -78.486194 | 13 years | Male | Skin | Unidentified unit |
| H1 | 15-0690 | -0.202333 -78.486194 | 5 years | Male | Tracheal aspirates | Unidentified unit |
| H1 | 15-0691 | -0.202333 -78.486194 | 5 months | Female | Tracheal aspirates | Unidentified unit |
| H1 | 15-0780 | -0.202333 -78.486194 | 13 years | Female | Blood | General surgery |
| H2 | 15-0795 | -0.127778 -78.49750 | 82 years | Male | Sputum | Unidentified |
| H2 | 15-0853 | -0.127778 -78.49750 | 37 years | Female | Abscesses | Traumatology |
| H1 | 15-0900 | -0.202333 -78.486194 | 13 years | Male | Sputum | General surgery |
| H1 | 15-0923 | -0.202333 -78.486194 | 4 years | Female | Tracheal aspirates | Intensive care unit |
| H1 | 15-0927 | -0.202333 -78.486194 | NA | NA | Unidentified | Intensive care unit |
| H1 | 15-0973 | -0.202333 -78.486194 | 4 months | Female | Catheters | Neonatal unit |
| H1 | 15-0985 | -0.202333 -78.486194 | 5 months | Female | Tracheal aspirates | Intensive care unit |
| H1 | 15-0989 | -0.202333 -78.486194 | 1 year | Male | Tracheal aspirates | Intensive care unit |
| H1 | 15-0997 | -0.202333 -78.486194 | 9 years | Female | Pleural fluid | Cardiology |
| H1 | 15-1004 | -0.202333 -78.486194 | 1 year | Male | Abscesses | Traumatology |
| H1 | 15-1064 | -0.202333 -78.486194 | 6 years | Male | Sputum | Infectious diseases |
| H1 | 15-1108 | -0.202333 -78.486194 | 6 years | Female | Skin | Burn unit |
| H1 | 15-1109 | -0.202333 -78.486194 | 13 years | Male | Hip wound | Traumatology |
| H1 | 15-1119 | -0.202333 -78.486194 | 3 years | Female | Tracheal aspirates | Intensive care unit |
| H1 | 15-1138 | -0.202333 -78.486194 | 3 months | Female | Wounds | Pediatric unit |
| H1 | 15-1139 | -0.202333 -78.486194 | 2 years | Male | Cerebrospinal fluid | Intensive care unit |
| H3 | 15-1175 | -0.24425 -78.541083 | 78 years | Male | Tracheal aspirates | Intensive care unit |
| H3 | 15-1352 | -0.24425 -78.541083 | 42 years | Female | Tracheal aspirates | Intensive care unit |
| H4 | 15-1367 | -0.089611 -78.476444 | 66 years | Female | Wounds | External consultation |
| H4 | 15-1368 | -0.089611 -78.476444 | 37 years | Female | Abscesses | External consultation |

**Continue…**

| **Hospital/River code** | **Isolated code** | **Geographical Coordinates** | **Age** | **Sex** | **Sample type** | **Hospital units/River name** |
| --- | --- | --- | --- | --- | --- | --- |
| RMO1 | 16-RMO1 | -0.090268 -78.487035 | NA | NA | NA | Machángara River |
| RMO2 | 16-RMO2 | -0.05142 -78.45193 | NA | NA | NA | Machángara River |
| RMO3 | 16-RMO3 | -0.05704 -78.45284 | NA | NA | NA | Machángara River |
| RMO4 | 16-RMO4 | -0.03986 -78.45027 | NA | NA | NA | Machángara River |
| RMO5 | 16-RMO5 | -0.03072 -78.44983 | NA | NA | NA | Machángara River |
| RMO6 | 16-RMO6 | -0.01246 -78.44088 | NA | NA | NA | Machángara River |
| RS1 | 16-RS1 | -0.37205 -78.506627 | NA | NA | NA | San Pedro River |
| RS2 | 16-RS2 | -0.329328 -78.459722 | NA | NA | NA | San Pedro River |
| RS3 | 16-RS3 | -0.323756 -78.458767 | NA | NA | NA | San Pedro River |
| RS4 | 16-RS4 | -0.303530 -78.460164 | NA | NA | NA | San Pedro River |
| RS5 | 16-RS5 | -0.30968 -78.45405 | NA | NA | NA | San Pedro River |
| RS6 | 16-RS6 | -0.316190 -78.456690 | NA | NA | NA | San Pedro River |
| RMA1 | 16-RMA1 | -0.18534 -78.41715 | NA | NA | NA | Monjas River |
| RMA2 | 16-RMA2 | -0.20905 -78.47659 | NA | NA | NA | Monjas River |
| RMA3 | 16-RMA3 | -0.22196 -78.48650 | NA | NA | NA | Monjas River |
| RMA4 | 16-RMA4 | -0.24386 -78.52479 | NA | NA | NA | Monjas River |
| RMA5 | 16-RMA5 | -0.25095 -78.52441 | NA | NA | NA | Monjas River |
| RMA6 | 16-RMA6 | -0.25688 -78.52592 | NA | NA | NA | Monjas River |
| RP1 | 16-RP1 | -0.309327 -78.443223 | NA | NA | NA | Pita River |
| RP2 | 16-RP2 | -0.311571 -78.439700 | NA | NA | NA | Pita River |
| RP3 | 16-RP3 | -0.316030 -78.433596 | NA | NA | NA | Pita River |
| RP4 | 16-RP4 | -0.319200 -78.431344 | NA | NA | NA | Pita River |
| RP5 | 16-RP5 | -0.34867 -78.413672 | NA | NA | NA | Pita River |
| RP6 | 16-RP6 | -0.35605 -78.40988 | NA | NA | NA | Pita River |
| RG1 | 16-RG1 | -0.071655 -78.3749 | NA | NA | NA | Guayllabamba River |
| RG2 | 16-RG2 | -0.065041 -78.374175 | NA | NA | NA | Guayllabamba River |
| RG3 | 16-RG3 | -0.06862 -78.37289 | NA | NA | NA | Guayllabamba River |
