# Supplementary material for: Carbapenemase genes in clinical and environmental isolates of Acinetobacter spp. from Quito, Ecuador
Source: PeerJ. 2024 Apr 25;12:e17199. doi: 10.7717/peerj.17199 (PMC11056107; doi:10.7717/peerj.17199)
Supplement: Supplemental Information 2 — NA: Not applied, Aba: A. baumannii, Aha: A. haemolyticus, ABC: A. baumannii/calcoaceticus complex, ng/µl: nanograme/microlitre, Y: yes, N: not. Biochemical results are expressed as positive (+) and negative (−). The antibiotic susceptibility profile are expresed as Intermediate (I), Sensistive (S), and Resistance (R). [file peerj-12-17199-s002.docx]

| **Sample ID** | **Biochemical test** | | | **Monodisc test** | **Diffusion test Antibiotics** | | | | | | | | | | | | | | | | | | | | | |
| --- | --- | --- | --- | --- | --- | --- | --- | --- | --- | --- | --- | --- | --- | --- | --- | --- | --- | --- | --- | --- | --- | --- | --- | --- | --- | --- |
|  | **Oxidase** | **Catalase** | **Vitek** | **EDTA** | **MEM** | | **IPM** | | **TOB** | | **SAM** | | **CAZ** | | **CIP** | | **GEN** | | **SXT** | | **AMK** | | **FEP** | | **TZP** | |
| 15-0014 | - | + | NA | - | 6 | R | 8 | R | 16 | S | 6 | R | 6 | R | 6 | R | 11 | R | 6 | R | 6 | R | 8 | R | 8 | R |
| 15-0107 | - | + | NA | - | 7 | R | 8 | R | 15 | S | 7 | R | 6 | R | 6 | R | 10 | R | 6 | R | 7 | R | 8 | R | 8 | R |
| 15-0115 | - | + | NA | - | 9 | R | 11 | R | 12 | R | 12 | I | 7 | R | 6 | R | 6 | R | 6 | R | 11 | R | 9 | R | 9 | R |
| 15-0117 | - | + | NA | - | 7 | R | 8 | R | 14 | I | 8 | R | 6 | R | 6 | R | 11 | R | 6 | R | 8 | R | 6 | R | 7 | R |
| 15-0122 | - | + | NA | - | 6 | R | 7 | R | 7 | R | 6 | R | 12 | R | 6 | R | 6 | R | 6 | R | 9 | R | 6 | R | 7 | R |
| 15-0176 | - | + | NA | - | 6 | R | 7 | R | 7 | R | 7 | R | 12 | R | 6 | R | 6 | R | 6 | R | 9 | R | 6 | R | 7 | R |
| 15-0181 | - | + | NA | - | 9 | R | 11 | R | 11 | R | 11 | R | 7 | R | 6 | R | 6 | R | 6 | R | 11 | R | 9 | R | 8 | R |
| 15-0184 | - | + | NA | - | 8 | R | 9 | R | 14 | I | 8 | R | 7 | R | 6 | R | 11 | R | 6 | R | 14 | R | 9 | R | 8 | R |
| 15-0252 | - | + | NA | - | 7 | R | 8 | R | 14 | I | 9 | R | 6 | R | 6 | R | 11 | R | 6 | R | 13 | R | 8 | R | 7 | R |
| 15-0255 | - | + | NA | - | 8 | R | 9 | R | 16 | S | 9 | R | 6 | R | 6 | R | 12 | R | 6 | R | 12 | R | 9 | R | 8 | R |
| 15-0259 | - | + | NA | - | 7 | R | 8 | R | 14 | I | 9 | R | 6 | R | 6 | R | 11 | R | 6 | R | 13 | R | 8 | R | 7 | R |
| 15-0499 | - | + | NA | - | 8 | R | 9 | R | 16 | S | 9 | R | 6 | R | 6 | R | 11 | R | 6 | R | 13 | R | 9 | R | 7 | R |
| 15-0500 | - | + | NA | - | 8 | R | 8 | R | 14 | I | 9 | R | 6 | R | 6 | R | 11 | R | 6 | R | 14 | R | 9 | R | 8 | R |
| 15-0502 | - | + | NA | - | 8 | R | 9 | R | 13 | I | 7 | R | 6 | R | 6 | R | 12 | R | 6 | R | 12 | R | 8 | R | 6 | R |
| 15-0540 | - | + | NA | - | 8 | R | 9 | R | 11 | R | 10 | R | 6 | R | 6 | R | 12 | R | 6 | R | 13 | R | 9 | R | 8 | R |
| 15-0577 | - | + | NA | - | 12 | R | 12 | R | 13 | I | 6 | R | 14 | R | 14 | R | 12 | R | 19 | S | 13 | R | 13 | R | 10 | R |
| 15-0578 | - | + | NA | - | 8 | R | 9 | R | 14 | I | 9 | R | 6 | R | 6 | R | 11 | R | 6 | R | 12 | R | 9 | R | 8 | R |
| 15-0579 | - | + | NA | - | 7 | R | 8 | R | 13 | I | 9 | R | 6 | R | 6 | R | 11 | R | 6 | R | 13 | R | 9 | R | 7 | R |
| 15-0580 | - | + | NA | - | 8 | R | 9 | R | 16 | S | 9 | R | 6 | R | 6 | R | 12 | R | 6 | R | 13 | R | 9 | R | 7 | R |
| 15-0581 | - | + | NA | - | 7 | R | 8 | R | 14 | I | 8 | R | 6 | R | 6 | R | 10 | R | 6 | R | 7 | R | 7 | R | 8 | R |
| 15-0582 | - | + | NA | - | 8 | R | 9 | R | 16 | S | 9 | R | 6 | R | 6 | R | 13 | I | 6 | R | 14 | R | 9 | R | 7 | R |
| 15-0589 | - | + | NA | - | 7 | R | 8 | R | 13 | I | 10 | R | 6 | R | 6 | R | 11 | R | 6 | R | 11 | R | 9 | R | 7 | R |

**Continue…**

| **Sample ID** | **Biochemical test** | | | **Monodisc test** | **Diffusion test Antibiotics** | | | | | | | | | | | | | | | | | | | | | |
| --- | --- | --- | --- | --- | --- | --- | --- | --- | --- | --- | --- | --- | --- | --- | --- | --- | --- | --- | --- | --- | --- | --- | --- | --- | --- | --- |
|  | **Oxidase** | **Catalase** | **Vitek** | **EDTA** | **MEM** | | **IPM** | | **TOB** | | **SAM** | | **CAZ** | | **CIP** | | **GEN** | | **SXT** | | **AMK** | | **FEP** | | **TZP** | |
| 15-0590 | - | + | NA | - | 8 | R | 9 | R | 8 | R | 7 | R | 14 | R | 6 | R | 6 | R | 6 | R | 9 | R | 9 | R | 9 | R |
| 15-0591 | - | + | NA | - | 7 | R | 8 | R | 15 | S | 8 | R | 6 | R | 6 | R | 11 | R | 6 | R | 7 | R | 6 | R | 7 | R |
| 15-0608 | - | + | NA | - | 8 | R | 9 | R | 7 | R | 8 | R | 13 | R | 6 | R | 6 | R | 6 | R | 9 | R | 9 | R | 8 | R |
| 15-0610 | - | + | NA | - | 8 | R | 8 | R | 6 | R | 9 | R | 6 | R | 6 | R | 11 | R | 6 | R | 11 | R | 9 | R | 7 | R |
| 15-0659 | - | + | NA | - | 7 | R | 7 | R | 11 | R | 6 | R | 6 | R | 26 | S | 21 | S | 24 | S | 18 | S | 6 | R | 9 | R |
| 15-0669 | - | + | NA | - | 8 | R | 9 | R | 1 | R | 9 | R | 6 | R | 6 | R | 12 | R | 6 | R | 13 | R | 9 | R | 8 | R |
| 15-0687 | - | + | NA | - | 8 | R | 9 | R | 16 | S | 9 | R | 6 | R | 6 | R | 13 | I | 6 | R | 11 | R | 9 | R | 8 | R |
| 15-0690 | - | + | NA | - | 9 | R | 10 | R | 16 | S | 10 | R | 6 | R | 6 | R | 11 | R | 6 | R | 11 | R | 10 | R | 8 | R |
| 15-0691 | - | + | NA | - | 8 | R | 9 | R | 16 | S | 10 | R | 6 | R | 6 | R | 12 | R | 9 | R | 15 | I | 9 | R | 7 | R |
| 15-0780 | - | + | NA | - | 7 | R | 8 | R | 15 | S | 9 | R | 6 | R | 6 | R | 11 | R | 6 | R | 8 | R | 6 | R | 8 | R |
| 15-0795 | - | + | NA | - | 7 | R | 8 | R | 15 | S | 8 | R | 6 | R | 6 | R | 11 | R | 6 | R | 8 | R | 6 | R | 8 | R |
| 15-0853 | - | + | NA | - | 8 | R | 11 | R | 8 | R | 11 | R | 12 | R | 16 | I | 6 | R | 6 | R | 9 | R | 10 | R | 9 | R |
| 15-0900 | - | + | NA | - | 8 | R | 6 | R | 18 | S | 13 | I | 19 | S | 15 | R | 13 | I | 12 | I | 16 | I | 13 | R | 15 | R |
| 15-0923 | - | + | NA | - | 7 | R | 8 | R | 15 | S | 8 | R | 6 | R | 6 | R | 11 | R | 6 | R | 6 | R | 7 | R | 7 | R |
| 15-0927 | - | + | NA | - | 8 | R | 9 | R | 16 | S | 9 | R | 6 | R | 6 | R | 11 | R | 6 | R | 6 | R | 8 | R | 9 | R |
| 15-0973 | - | + | NA | - | 7 | R | 8 | R | 15 | S | 10 | R | 6 | R | 6 | R | 11 | R | 6 | R | 8 | R | 6 | R | 7 | R |
| 15-0985 | - | + | NA | - | 6 | R | 8 | R | 14 | I | 8 | R | 6 | R | 6 | R | 10 | R | 6 | R | 8 | R | 6 | R | 7 | R |
| 15-0989 | - | + | NA | - | 7 | R | 8 | R | 15 | S | 8 | R | 6 | R | 6 | R | 11 | R | 6 | R | 7 | R | 7 | R | 7 | R |
| 15-0997 | - | + | NA | - | 7 | R | 7 | R | 16 | S | 9 | R | 6 | R | 6 | R | 17 | S | 6 | R | 8 | R | 8 | R | 8 | R |
| 15-1004 | - | + | NA | - | 8 | R | 9 | R | 15 | S | 8 | R | 6 | R | 6 | R | 11 | R | 6 | R | 7 | R | 7 | R | 6 | R |

**Continue…**

| **Sample ID** | **Biochemical test** | | | **Monodisc test** | **Diffusion test Antibiotics** | | | | | | | | | | | | | | | | | | | | | |
| --- | --- | --- | --- | --- | --- | --- | --- | --- | --- | --- | --- | --- | --- | --- | --- | --- | --- | --- | --- | --- | --- | --- | --- | --- | --- | --- |
|  | **Oxidase** | **Catalase** | **Vitek** | **EDTA** | **MEM** | | **IPM** | | **TOB** | | **SAM** | | **CAZ** | | **CIP** | | **GEN** | | **SXT** | | **AMK** | | **FEP** | | **TZP** | |
| 15-1064 | - | + | NA | - | 7 | R | 8 | R | 16 | S | 9 | R | 6 | R | 6 | R | 10 | R | 6 | R | 8 | R | 8 | R | 7 | R |
| 15-1108 | - | + | NA | - | 6 | R | 9 | R | 18 | S | 7 | R | 6 | R | 6 | R | 12 | R | 6 | R | 8 | R | 8 | R | 8 | R |
| 15-1109 | - | + | NA | - | 7 | R | 9 | R | 14 | I | 7 | R | 6 | R | 6 | R | 8 | R | 6 | R | 6 | R | 7 | R | 8 | R |
| 15-1119 | - | + | NA | - | 7 | R | 8 | R | 16 | S | 7 | R | 6 | R | 6 | R | 6 | R | 6 | R | 7 | R | 7 | R | 7 | R |
| 15-1138 | - | + | NA | - | 12 | R | 17 | R | 15 | S | 15 | S | 19 | S | 20 | I | 15 | S | 17 | S | 15 | I | 21 | S | 17 | R |
| 15-1139 | - | + | NA | - | 7 | R | 9 | R | 17 | S | 8 | R | 6 | R | 6 | R | 17 | S | 6 | R | 6 | R | 9 | R | 8 | R |
| 15-1175 | - | + | NA | - | 7 | R | 8 | R | 14 | I | 8 | R | 6 | R | 6 | R | 10 | R | 6 | R | 8 | R | 6 | R | 7 | R |
| 15-1352 | - | + | NA | - | 7 | R | 8 | R | 15 | S | 8 | R | 6 | R | 6 | R | 10 | R | 6 | R | 7 | R | 6 | R | 8 | R |
| 15-1367 | - | + | NA | - | 7 | R | 8 | R | 14 | I | 8 | R | 6 | R | 6 | R | 11 | R | 6 | R | 8 | R | 6 | R | 8 | R |
| 15-1368 | - | + | NA | - | 7 | R | 8 | R | 14 | I | 9 | R | 6 | R | 6 | R | 11 | R | 6 | R | 8 | R | 6 | R | 8 | R |
| 16-RS3a | - | + | *Aba* | - | 16 | I | 19 | I | 16 | S | 18 | S | 15 | I | 17 | I | 16 | S | 16 | S | 14 | R | 16 | I | 16 | R |
| 16-RS3b | - | + | *Aba* | - | 16 | I | 20 | I | 16 | S | 17 | S | 16 | I | 17 | I | 15 | S | 16 | S | 14 | R | 16 | I | 16 | R |
| 16-RMA3a | - | + | *Aha* | - | 15 | I | 18 | R | 16 | S | 18 | S | 13 | R | 17 | I | 14 | I | 13 | I | 13 | R | 15 | I | 16 | R |
| 16-RMA3b | - | + | *Aba* | - | 16 | I | 18 | R | 17 | S | 18 | S | 15 | I | 18 | I | 15 | S | 14 | I | 15 | I | 16 | I | 16 | R |
| 16-RMA3c | - | + | *Aba* | - | 17 | I | 20 | I | 17 | S | 20 | S | 16 | I | 19 | I | 16 | S | 17 | S | 15 | I | 16 | I | 15 | R |
| 16-RMA2 | - | + | *Aba* | - | 16 | I | 18 | R | 16 | S | 18 | S | 15 | I | 18 | I | 15 | S | 14 | I | 15 | I | 16 | I | 17 | R |
| 16-RP2 | - | + | *Aba* | - | 18 | S | 21 | I | 16 | S | 17 | S | 16 | I | 18 | I | 16 | S | 16 | S | 15 | I | 16 | I | 17 | R |
| 16-RMO1a | - | + | ABC | - | 16 | I | 17 | R | 17 | S | 18 | S | 13 | R | 16 | I | 15 | S | 15 | I | 15 | I | 15 | I | 16 | R |
| 16-RMO1b | - | + | ABC | - | 17 | I | 20 | I | 17 | S | 20 | S | 16 | I | 18 | I | 16 | S | 16 | S | 16 | I | 17 | I | 17 | R |
| 16-RMO2 | - | + | ABC | - | 17 | I | 18 | R | 16 | S | 17 | S | 13 | R | 18 | I | 15 | S | 14 | I | 16 | I | 14 | R | 16 | R |
