# Supplementary material for: Carbapenemase genes in clinical and environmental isolates of Acinetobacter spp. from Quito, Ecuador
Source: PeerJ. 2024 Apr 25;12:e17199. doi: 10.7717/peerj.17199 (PMC11056107; doi:10.7717/peerj.17199)
Supplement: Supplemental Information 3 — A. baumannii was identified by the amplification of both products, 490 bp and 294 bp, and A. pittii with a product of 194 bp of gyrB gene. The species identification was corroborate by sequencing on partial rpo gene. PCR results are expressed for carbapenen resistance genes as positive (+) and negative (−). The table details the GenBank number acccess of sequences obtained in this study. [file peerj-12-17199-s003.docx]

| **Isolated code** | **DNA concentration (ng/µl)** | **Species identification** | **Carbapenems-resistant genes (*bla*)** | | | | | | | | **GenBank accession numbers** | | | | |
| --- | --- | --- | --- | --- | --- | --- | --- | --- | --- | --- | --- | --- | --- | --- | --- |
|  |  |  | ***_SIM_*** | ***_GIM_*** | ***_SPM_*** | ***_GES_*** | ***_OXA-51_*** | ***_OXA-23_*** | ***_OXA-24_*** | ***_OXA-143_*** | **OXA-65** | **OXA-70** | **OXA-23** | **OXA-366** | **OXA-72** |
| 15-0014 | 65.7 | *A. baumannii* | - | - | - | - | + | + | - | - | OP554146 | - | OP554169 | - | - |
| 15-0107 | 97.1 | *A. baumannii* | - | - | - | - | + | + | - | - | - | - | MF594755 | - | - |
| 15-0115 | 183 | *A. baumannii* | - | - | - | - | + | + | - | - | OP554147 | - | OP554170 | - | - |
| 15-0117 | 89.1 | *A. baumannii* | - | - | - | - | + | + | - | - | MF594725 | - | MF594756 | - | - |
| 15-0122 | 88.7 | *A. baumannii* | - | - | + | - | + | + | - | - | MF594726 | - | - | MF594757 | - |
| 15-0176 | 62.5 | *A. baumannii* | - | - | - | - | + | + | - | - | OP554148 | - | - | OP554171 | - |
| 15-0181 | 86.6 | *A. baumannii* | - | - | - | - | + | + | - | - | MF594727 | - | MF594758 | - | - |
| 15-0184 | 88.5 | *A. baumannii* | - | - | - | - | + | + | - | - | - | - | - | - | - |
| 15-0252 | 67 | *A. baumannii* | - | - | - | - | + | + | - | - | OP554149 | - | OP554172 | - | - |
| 15-0255 | 62.8 | *A. baumannii* | - | - | - | - | + | + | - | - | OP554150 | - | OP554173 | - | - |
| 15-0259 | 134.5 | *A. baumannii* | - | - | - | - | + | + | - | - | OP554151 | - | OP554174 | - | - |
| 15-0499 | 93.4 | *A. baumannii* | - | - | - | - | + | + | - | - | MF594728 | - | MF594759 | - | - |
| 15-0500 | 112.3 | *A. baumannii* | - | - | - | - | + | + | - | - | MF594729 | - | MF594760 | - | - |
| 15-0502 | 340.8 | *A. baumannii* | - | - | - | - | + | + | - | - | OP554152 | - | OP554175 | - | - |
| 15-0540 | 81.1 | *A. baumannii* | - | - | - | - | + | + | - | - | OP554153 | - | OP554176 | - | - |
| 15-0577 | 79 | *A. baumannii* | - | - | - | - | + | + | - | - | MF594730 | - | MF594761 | - | - |
| 15-0578 | 66.5 | *A. baumannii* | - | - | - | - | + | + | - | - | MF594742 | - | MF594762 | - | - |
| 15-0579 | 104 | *A. baumannii* | - | - | - | - | + | + | - | - | OP554154 | - | OP554177 | - | - |
| 15-0580 | 122.1 | *A. baumannii* | - | - | - | - | + | + | - | - | MF594731 | - | MF594763 | - | - |
| 15-0581 | 57.4 | *A. baumannii* | - | - | - | - | + | + | - | - | MF594743 | - | MF594764 | - | - |
| 15-0582 | 50 | *A. baumannii* | - | - | - | - | + | + | - | - | MF594744 | - | MF594765 | - | - |
| 15-0589 | 60.6 | *A. baumannii* | - | - | - | - | + | + | - | - | MF594732 | - | MF594766 | - | - |
| 15-0590 | 64.5 | *A. baumannii* | - | - | - | - | + | + | - | - | MF594733 | - | MF594767 | - | - |

**Continue…**

| **Isolated code** | **DNA concentration (ng/µl)** | **Species identification** | **Carbapenems-resistant genes (*bla*)** | | | | | | | | **GenBank accession numbers** | | | | |
| --- | --- | --- | --- | --- | --- | --- | --- | --- | --- | --- | --- | --- | --- | --- | --- |
|  |  |  | ***_SIM_*** | ***_GIM_*** | ***_SPM_*** | ***_GES_*** | ***_OXA-51_*** | ***_OXA-23_*** | ***_OXA-24_*** | ***_OXA-143_*** | **OXA-65** | **OXA-70** | **OXA-23** | **OXA-366** | **OXA-72** |
| 15-0591 | 126.1 | *A. baumannii* | - | - | + | - | + | + | - | - | MF594734 | - | MF594768 | - | - |
| 15-0608 | 81.6 | *A. baumannii* | - | - | - | - | + | + | - | - | OP554155 | - | OP554178 | - | - |
| 15-0610 | 64.1 | *A. baumannii* | - | - | - | - | + | + | - | - | MF594735 | - | MF594769 | - | - |
| 15-0659 | 432.3 | *A. baumannii* | - | - | - | - | + | + | + | - | OP554156 | - | - | OP554179 | MF594778 |
| 15-0669 | 395.8 | *A. baumannii* | - | - | - | + | + | + | - | - | OP554157 | - | OP554180 | - | - |
| 15-0687 | 105.6 | *A. baumannii* | - | - | - | - | + | + | - | - | OP554158 | - | OP554181 | - | - |
| 15-0690 | 118.6 | *A. baumannii* | - | - | + | + | + | + | - | - | OP554159 | - | OP554182 | - | - |
| 15-0691 | 244.5 | *A. pittii* | - | - | - | + | + | + | + | - | - | - | - | OP554183 | MF594781 |
| 15-0780 | 430.3 | *A. baumannii* | - | - | + | - | + | + | - | - | OP554160 | - | - | OP554184 | - |
| 15-0795 | 160.6 | *A. baumannii* | - | - | + | - | + | + | - | - | MF594736 | - | MF594770 | - | - |
| 15-0853 | -4.6 | *A. baumannii* | - | - | + | + | + | + | - | - | MF594737 | - | MF594771 | - | - |
| 15-0900 | 420.9 | *A. baumannii* | - | - | - | + | + | + | - | - | - | - | - | - | - |
| 15-0923 | 142.9 | *A. baumannii* | - | - | - | - | + | + | - | - | OP554161 | - | OP554185 | - | - |
| 15-0927 | 139.8 | *A. baumannii* | - | - | - | - | + | + | - | - | OP554162 | - | OP554186 | - | - |
| 15-0973 | 15.5 | *A. baumannii* | - | - | - | - | + | + | - | - | OP554163 | - | OP554187 | - | - |
| 15-0985 | 79.1 | *A. baumannii* | - | - | - | - | + | + | + | - | OP554164 | - | - | OP554188 | MF594779 |
| 15-0989 | 58 | *A. baumannii* | - | - | - | - | + | + | - | - | OP554165 | - | OP554189 | - | - |
| 15-0997 | 220.1 | *A. baumannii* | - | - | - | - | + | + | - | - | - | - | - | OP554190 | - |
| 15-1004 | 133.2 | *A. baumannii* | - | - | - | - | + | + | - | - | MF594745 | - | MF594772 | - | - |
| 15-1064 | 65.3 | *A. baumannii* | - | - | - | - | + | + | + | - | MF594738 | - | MF594777 | - | MF594783 |
| 15-1108 | 440.6 | *A. baumannii* | - | - | - | - | + | + | - | - | - | - | - | - | - |
| 15-1109 | 62 | *A. baumannii* | - | - | - | - | + | + | - | - | OP554166 | - | OP554191 | - | - |

**Continue…**

| **Isolated code** | **DNA concentration (ng/µl)** | **Species identification** | **Carbapenems-resistant genes (*bla*)** | | | | | | | | **GenBank accession numbers** | | | | |
| --- | --- | --- | --- | --- | --- | --- | --- | --- | --- | --- | --- | --- | --- | --- | --- |
|  |  |  | ***_SIM_*** | ***_GIM_*** | ***_SPM_*** | ***_GES_*** | ***_OXA-51_*** | ***_OXA-23_*** | ***_OXA-24_*** | ***_OXA-143_*** | **OXA-65** | **OXA-70** | **OXA-23** | **OXA-366** | **OXA-72** |
| 15-1119 | 229.2 | *A. baumannii* | - | - | - | - | + | + | - | - | OP554167 | - | OP554192 | - | - |
| 15-1138 | 295.8 | *A. baumannii* | - | - | + | - | + | + | - | - | MF594739 | - | MF594773 | - | - |
| 15-1139 | 163.1 | *A. baumannii* | - | - | - | - | + | + | - | - | OP554168 | - | OP554193 | - | - |
| 15-1175 | 312.2 | *A. pittii* | + | - | - | + | - | - | + | + | - | - | - | - | MF594782 |
| 15-1352 | 406.9 | *A. baumannii* | - | - | - | - | + | + | - | - | - | MF594747 | - | MF594774 | - |
| 15-1367 | 125.1 | *A. baumannii* | - | - | - | - | + | + | + | - | - | MF594740 | - | MF594775 | MF594780 |
| 15-1368 | 166.6 | *A. baumannii* | - | - | - | - | + | + | - | - | MF594741 | - | - | MF594776 | - |
| 16-RS3a | 268.2 | *A. baumannii* | - | - | - | - | + | - | - | - | MF594748 | - | - | - | - |
| 16-RS3b | 179 | *A. baumannii* | - | - | - | - | + | - | - | - | MF594749 | - | - | - | - |
| 16-RMA3a | 325.6 | *A. baumannii* | - | - | - | - | + | - | - | - | - | - | - | - | - |
| 16-RMA3b | 214.5 | *A. baumannii* | - | - | - | - | + | - | - | - | - | - | - | - | - |
| 16-RMA3c | 154.5 | *A. baumannii* | - | - | - | - | + | - | - | - | MF594750 | - | - | - | - |
| 16-RMA2 | 371.8 | *A. baumannii* | - | - | - | - | + | - | - | - |  | - | - | - | - |
| 16-RP2 | 330 | *A. baumannii* | - | - | - | - | + | - | - | - | MF594751 | - | - | - | - |
| 16-RMO1a | 321.8 | *A. baumannii* | - | - | - | - | - | - | - | - | - | - | - | - | - |
| 16-RMO1b | 230.1 | *A. baumannii* | - | - | - | - | - | - | - | - | - | - | - | - | - |
| 16-RMO2 | 286.8 | *A. baumannii* | - | - | - | - | - | - | - | - | - | - | - | - | - |
